# Non-steroidal anti-inflammatory drugs and bladder cancer prevention

**DOI:** 10.1054/bjoc.1999.1106

**Published:** 2000-03-06

**Authors:** J E Castelao, J-M Yuan, M Gago-Dominguez, M C Yu, R K Ross

**Affiliations:** Department of Preventive Medicine, USC/Norris Comprehensive Cancer Center, M/S #44, Keck University of Southern California School of Medicine, 1441 Eastlake Avenue, Los Angeles, CA, 90033-0800, USA

**Keywords:** bladder cancer, analgesics, aspirin, acetaminophen, phenacetin, other NSAID drugs

## Abstract

Inclusion of phenacetin among ‘proven’ human carcinogens by the IARC in 1987, raised concerns about the carcinogenic potential of acetaminophen, its major metabolite. Acetaminophen has been implicated as a possible causal agent in the development of cancer of the renal pelvis. The bladder and renal pelvis, which derive from the same embryological structure, share the same transitional type of epithelium. Past studies have been inconclusive on the possible relationship among these analgesics and bladder cancer but no large, highly detailed study of this association has been conducted. A population-based case–control study conducted in Los Angeles, California, involved 1514 incident bladder cancer cases and an equal number of controls who were matched to the index cases by sex, date of birth (within 5 years) and race. Detailed information on medication use and prior medical conditions was collected through in-person interviews. Regular use of analgesics was not associated with an increased risk of bladder cancer in either men or women. In fact, compared with non- or irregular users, regular analgesic users were at a decreased risk of bladder cancer overall (odds ratio (OR) = 0.81, 95% confidence interval (CI) = 0.68–0.96). However, there were clear differences in both the direction and strength of the associations between the different formulation classes of analgesics and bladder cancer risk. Intake of phenacetin was positively related to bladder cancer risk in a dose-dependent manner while intake of its major metabolite in humans, acetaminophen, was unrelated to risk. Intake of all classes of NSAIDs, except pyrazolon derivatives, were negatively associated with bladder cancer risk, with suggestive evidence that the protective effect varies in strength by subcategories of formulation. Acetic acids seemed to exhibit the strongest protective effect, whereas aspirin/other salicylic acids and oxicam showed the weakest protection. © 2000 Cancer Research Campaign


British Journal of Cancer (2000) **82**(7), 1364–1369
© 2000 Cancer Research Campaign
DOI: 10.1054/ bjoc.1999.1106, available online at http://www.idealibrary.com on IDEAL®

### Non-steroidal anti-inflammatory drugs and bladder cancer prevention

**JE Castelao, J-M Yuan, M Gago-Dominguez, MC Yu and RK Ross**

Department of Preventive Medicine, USC/Norris Comprehensive Cancer Center, M/S #44, Keck University of Southern California School of Medicine, 1441 Eastlake Avenue, Los Angeles, CA 90033-0800, USA

**Summary** Inclusion of phenacetin among 'proven' human carcinogens by the IARC in 1987, raised concerns about the carcinogenic potential of acetaminophen, its major metabolite. Acetaminophen has been implicated as a possible causal agent in the development of cancer of the renal pelvis. The bladder and renal pelvis, which derive from the same embryological structure, share the same transitional type of epithelium. Past studies have been inconclusive on the possible relationship among these analgesics and bladder cancer but no large, highly detailed study of this association has been conducted. A population-based case–control study conducted in Los Angeles, California, involved 1514 incident bladder cancer cases and an equal number of controls who were matched to the index cases by sex, date of birth (within 5 years) and race. Detailed information on medication use and prior medical conditions was collected through in-person interviews. Regular use of analgesics was not associated with an increased risk of bladder cancer in either men or women. In fact, compared with non- or irregular users, regular analgesic users were at a decreased risk of bladder cancer overall (odds ratio (OR) = 0.81, 95% confidence interval (CI) = 0.68–0.96). However, there were clear differences in both the direction and strength of the associations between the different formulation classes of analgesics and bladder cancer risk. Intake of phenacetin was positively related to bladder cancer risk in a dose-dependent manner while intake of its major metabolite in humans, acetaminophen, was unrelated to risk. Intake of all classes of NSAIDs, except pyrazolon derivatives, were negatively associated with bladder cancer risk, with suggestive evidence that the protective effect varies in strength by subcategories of formulation. Acetic acids seemed to exhibit the strongest protective effect, whereas aspirin/other salicylic acids and oxicam showed the weakest protection. © 2000 Cancer Research Campaign

**Keywords:** bladder cancer; analgesics; aspirin; acetaminophen; phenacetin; other NSAID drugs

In the USA, an estimated 51 200 cases of cancer of the bladder are diagnosed annually and 10 600 deaths from the disease occur each year (Boring et al, 1994). Bladder cancer currently accounts for 6% of all new cancer cases in men and 2% of new cancer cases in women. The most established aetiological risk factors for bladder cancer are cigarette smoking and occupational exposure to arylamines (Yu and Ross, 1998). Many other possible aetiological factors have been extensively explored (e.g. caffeine intake, use of artificial sweetener), but none have been definitively established as causative agents. A number of experimental and laboratory studies have examined the relationship between analgesic use and bladder cancer risk; the evidence tends to vary by class of formulation. Phenacetin-based analgesics have been known for a long time to cause cancer of the renal pelvis in humans (Hultengren et al, 1965; Angervall et al, 1969) and experimental data indicates that the compound is carcinogenic to the bladder as well (Johansson, 1981; Murai et al, 1993). Epidemiological data regarding bladder cancer risk are relatively sparse, but generally are in support of phenacetin as a human bladder carcinogen (Fokkens, 1979; McCredie et al, 1983; Piper et al, 1985; McCredie and Stewart, 1988). Acetaminophen use, a phenacetin metabolite, has been examined as a separate risk factor in a few epidemiological (Piper et al, 1985; McCredie et al, 1988; Derby et al, 1996) and experimental studies (Flaks et al, 1985; Hiraga et al, 1985). On balance, the evidence points towards acetaminophen as being non-carcinogenic to the bladder. Human data on bladder cancer and non-steroidal anti-inflammatory drugs (NSAIDs), including aspirin, are largely lacking. However, a number of rodent studies have shown that several classes of NSAIDs are powerful inhibitors of chemically-induced bladder cancer (Grubbs et al, 1993; Klän et al, 1993; Shibata et al, 1993; Rao et al, 1996), thus raising the possibility that NSAID intake might protect against bladder cancer development in humans.

In 1986, we initiated a large-scale case–control study of bladder cancer in Los Angeles County with one of the primary aims being the investigation of chronic use of over-the-counter and prescription analgesics in relation to bladder cancer risk. We hypothesized that chronic use of analgesics was associated with increased bladder cancer risk. This report describes in detail our findings with respect to individual classes of analgesics by formulation.

*Received 7 April 1999*
*Revised 2 September 1999*
*Accepted 23 September 1999*

*Correspondence to:* JE Castelao

### MATERIALS AND METHODS

The Los Angeles County Cancer Surveillance Program (Bernstein and Ross, 1991), the population-based based Surveillance, Epidemiology and End Results (SEER) cancer registry of Los Angeles County, identified 2098 non-Asian patients aged 25–74 years with histologically confirmed bladder cancer between 1 January 1987 and 30 April 1996. Among these, 175 patients died before we could contact them or were too ill to be interviewed. Permission to contact 74 patients was denied by their physicians and 267 patients refused to be interviewed. Thus, we interviewed 75% (1582 out of 2098) of all eligible patients.

For each patient interviewed, we sought to recruit a control who was matched to the index case by sex, date of birth (within 5 years), race (non-Hispanic White, Hispanic, Black), and neighbourhood of residence at the time of cancer diagnosis. To search for these ‘neighbourhood’ controls, we followed a standard procedure that defines a sequence of houses on specified neighbourhood blocks. We attempted to identify the sex, age and race of all inhabitants of each housing unit; ‘not at home’ units were systematically revisited to complete the census. When we failed to find any resident who met our matching criteria after canvassing 150 housing units, we excluded race from the matching criteria. If a matched control based on this relaxed criteria could not be found within a maximum of 300 housing units, the case was dropped from the study. Sixty-eight cases were dropped from the study due to lack of a matched control, and 22 controls were not matched by race to the index case.

In-person, structured interviews were conducted in subjects’ homes. The questionnaire requested information up to 2 years prior to the diagnosis of cancer for cases and 2 years prior to diagnosis of cancer of the index case for matched controls. The questionnaire included information on demographic characteristics, height, weight, lifetime use of tobacco and alcohol, usual adult dietary habits, lifetime occupational history, prior medical conditions and prior use of medications.

In terms of analgesic use, we explicitly listed 44 over-the-counter and 33 prescription brand-name analgesics in the questionnaire (see Appendix). These brand-named drugs represent all common analgesics marketed in the USA since the 1950s. A picture album of the listed drugs was available to the respondent to assist in their recall of their usage. For each of the listed brand name analgesics, we first asked the subject whether they had ever taken the drug 20 or more times over their lifetime. If the answer was no, the subject was defined as a ‘non-user’. Otherwise, the subject was further asked if they had ever taken the drug two or more times a week for 1 month or longer. If the answer was no, the subject was classified as an ‘irregular user’. Otherwise, the subject was defined as a ‘regular user’ and was further asked about the ages at first and last use, duration of use, usual frequency and dosage of use, and the primary reason for such use. Aside from the 77 brand-name analgesics listed, the subject was asked if they had taken any other analgesics regularly. If the answer was yes, the names of the analgesic drugs were recorded, and ages at first and last use, duration of use, usual frequency and dosage of use, and the primary reason for use were similarly asked.

The formulations of each of the listed analgesics as well as those volunteered by study subjects were established through numerous pharmaceutical sources, including the annually updated Physician’s Desk Reference. Each active ingredient of brand-name analgesics was classified according to formulation as follows: aspirin, non-aspirin NSAIDs, acetaminophen, phenacetin and other. Similarly, each active ingredient of non-aspirin NSAIDs was further classified as propionic acid (such as ibuprofen and naproxen), acetic acid (such as indomethacin and sulindac), fenamic acid (such as meclofenamic), salicylic acid (such as magnesium salicylate and salicylamide), oxicam (such as piroxicam), and pyrazolon derivatives (such as phenylbutazone). Age-specific exposure to a given drug was estimated from the subject’s reported dose and duration of use at that age. Lifetime cumulative exposure (g) to a specific class of analgesics was computed by summing individual age-specific exposures across all ages and all brand-name drugs containing the active ingredient of interest. Cumulative exposures among regular users were grouped into tertiles or above/below median values according to their distributions among control subjects.

Data were analysed by standard matched-pair methods (Breslow and Day, 1980). The associations of bladder cancer with various exposure indices of analgesic use were measured by odds ratios (ORs) and their corresponding 95% confidence intervals (CIs) and *P*-values. Conditional logistic regression models were used to examine the relationship between analgesic use and bladder cancer risk with adjustment for other risk factors for bladder cancer (cigarette smoking and duration of employment as hairdresser/barber). Pairs in which either the case or the control failed to answer the relevant questions were eliminated from the corresponding analysis. ORs with 2-sided *P*-values less than 0.05 were considered statistically significant. All *P*-values quoted are 2-sided.

### RESULTS

The mean age of the patients at diagnosis of bladder cancer was 58 years. Most patients were non-Hispanic Whites ($n$ = 1413), with the remaining being Hispanic Whites ($n$ = 58), African-Americans ($n$ = 42) and Native Americans ($n$ = 1). On average, bladder cancer patients had a lower level of education than controls. The OR for bladder cancer was 0.56 (95% CI 0.48–0.67) for those who had attended college (13 years or more of schooling) compared with those who had a high school education or lower. Thus, all ORs presented below were adjusted for level of education (high school or less, college or above).

Relative to lifetime non-users of analgesics, irregular users of analgesics exhibited no increase in risk of bladder cancer (OR = 0.95, 95% CI 0.67–1.34), whereas regular users of analgesics (used at least twice a week for 1 month or longer) showed a 20% decrease in risk (OR = 0.81, 95% CI 0.68–0.96) relative to non- or irregular users after adjustment for cigarette smoking and duration of employment as hairdresser/barber (Table 1).

Table 2 presents the individual relationships between bladder cancer and the phenacetin-based and acetaminophen-based analgesics. After adjustment for cigarette smoking, duration of employment as hairdresser/barber and use of other analgesics, phenacetin use was related to a statistically non-significant 50% increase in bladder cancer risk that was, however, dose-dependent ($P$ = 0.03). The highest tertile of lifetime exposure was associated with a 1.8-fold increase in risk relative to non/irregular use of any analgesic. Acetaminophen use, on the other hand, was not related to increased risk (OR = 0.85, 95% CI 0.60–1.19), and there was no

**Table 1** Use of analgesics and risk of bladder cancer

| Use of any analgesic | Cases | Controls | OR[a] (95% CI) |
|---|---|---|---|
| Non/Irregular user | 961 | 920 | 1.0 |
| Regular user[b] | 553 | 594 | 0.81 (0.68–0.96) |

[a]Adjusted for level of education, current cigarette smoking status, number of cigarettes smoked per day, number of years of cigarette smoking, and number of years employed as hairdresser/barber. [b]Used at least twice a week for 1 month or longer.

© 2000 Cancer Research Campaign

**Table 2** Cumulative lifetime consumption of phenacetin- and acetaminophen-based analgesics in relation to risk of bladder cancer

| | Any users | | Exclusive users | |
|---|---|---|---|---|
| **Category of analgesic** | **Ca/Co** | **OR[a] (95% CI)** | **Ca/Co** | **OR[b] (95% CI)** |
| Phenacetin | | | | |
| Regular use | 82/64 | 1.52 (0.85–2.73) | – | – |
| Cumulative lifetime exposure (g) | | | | |
| < 46 | 25/18 | 1.35 (0.60–3.07) | – | – |
| 46–250 | 27/20 | 1.64 (0.73–3.70) | – | – |
| 251+ | 21/20 | 1.85 (0.77–4.43) | – | – |
| Acetaminophen | | | | |
| Regular use | 224/244 | 0.85 (0.60–1.19) | 60/50 | 1.03 (0.67–1.58) |
| Cumulative lifetime exposure (g) | | | | |
| < 114 | 62/75 | 0.75 (0.47–1.20) | 27/21 | 1.07 (0.56–2.04) |
| 114–885 | 57/82 | 0.68 (0.43–1.09) | 17/17 | 0.82 (0.39–1.72) |
| 886+ | 90/74 | 1.43 (0.87–2.35) | 15/7 | 1.93 (0.73–5.11) |

[a]Reference group = non/irregular users of analgesics. ORs after adjustment for education (less than college, college or above), current cigarette smoking status, number of cigarettes smoked per day, number of years of cigarette smoking, number of years employed as hairdresser/barber, NSAID use, and use of the other class of analgesics listed in the table. [b]ORs after adjustment for education (less than college, college or above), current cigarette smoking status, number of cigarettes smoked per day, number of years of cigarette smoking, and number of years employed as hairdresser/barber.

**Table 3** Cumulative lifetime consumption of various classes of NSAIDs in relation to risk of bladder cancer

| | Any users | | Exclusive users | |
|---|---|---|---|---|
| **Category of analgesic** | **Ca/Co** | **OR[a] (95% CI)** | **Ca/Co** | **OR[b] (95% CI)** |
| Aspirin | | | | |
| Regular use | 399/420 | 0.88 (0.70–1.12) | 175/187 | 0.85 (0.66–1.09) |
| Cumulative lifetime exposure (g) | | | | |
| < 245 | 156/139 | 0.99 (0.73–1.34) | 86/68 | 1.04 (0.72–1.49) |
| 245–1242 | 126/136 | 0.94 (0.68–1.30) | 61/67 | 0.92 (0.61–1.36) |
| 1243+ | 107/135 | 0.63 (0.43–0.92) | 25/48 | 0.49 (0.29–0.84) |
| Other salicylic acids | | | | |
| Regular use | 48/53 | 0.81 (0.45–1.45) | – | – |
| Cumulative lifetime exposure (g) | | | | |
| < 168 | 24/25 | 0.92 (0.43–1.97) | – | – |
| 168+ | 20/24 | 0.65 (0.31–1.37) | – | – |
| Acetic acids | | | | |
| Regular use | 30/54 | 0.54 (0.31–0.94) | 9/15 | 0.53 (0.21–1.36) |
| Cumulative lifetime exposure (g) | | | | |
| < 27 | 18/22 | 0.68 (0.31–1.47) | 6/3 | 1.05 (0.22–4.97) |
| 27+ | 10/25 | 0.46 (0.21–1.03) | 3/10 | 0.37 (0.09–1.49) |
| Propionic acids | | | | |
| Regular use | 131/151 | 0.70 (0.49–0.99) | 37/48 | 0.61 (0.37–0.99) |
| Cumulative lifetime exposure (g) | | | | |
| < 144 | 61/65 | 0.81 (0.51–1.28) | 21/24 | 0.75 (0.39–1.43) |
| 144+ | 63/79 | 0.55 (0.34–0.87) | 14/22 | 0.46 (0.22–0.98) |
| Oxicam | | | | |
| Regular use | 18/17 | 0.92 (0.42–2.02) | – | – |
| Pyrazolon derivatives | | | | |
| Regular use | 13/6 | 2.03 (0.68–6.07) | – | – |

[a]Reference group = non/irregular users of analgesics. ORs after adjustment for education (less than college, college or above), current cigarette smoking status, number of cigarettes smoked per day, number of years of cigarette smoking, number of years employed as hairdresser/barber, use of phenacetin, acetaminophen, and the other classes of analgesics listed in the table. [b]ORs after adjustment for education (less than college, college or above), current cigarette smoking status, number of cigarettes smoked per day, number of years of cigarette smoking, and number of years employed as hairdresser/barber.

dose–response relationship with lifetime exposure ($P$ = 0.80). Since use of different categories of analgesics are likely to be correlated, mutual adjustment for such highly correlated variables may not adequately control for the effect of confounding. One way of addressing this issue is to evaluate exclusive use of each type of analgesic. Table 2 shows that results based on exclusive use were consistent with those based on any use of acetaminophen. There were no exclusive users of phenacetin because all phenacetin-containing analgesics consumed by study subjects also contained aspirin.

Table 3 shows risk of bladder cancer by formulation subclass of NSAIDs. Except for pyrazolon derivatives, all other formulation subclasses of NSAIDs exhibited an inverse, dose-dependent association between intake and risk. However, the strength of the

 *© 2000 Cancer Research Campaign*

association seemed to vary by formulation. The strongest protective effect was observed with acetic acids (overall OR = 0.54) while aspirin/other salicylic acids (overall ORs of 0.88 and 0.81 respectively) and oxicam (overall OR = 0.92) showed the weakest protective effects. Propionic acids exhibited intermediate effects (overall OR about 0.70). When the analysis was confined to exclusive users of individual formulation subclasses of NSAIDs, results similar to those based on all subjects were obtained.

In contrast to the inverse exposure-risk relationship of other NSAIDs with bladder cancer, intake of pyrazolon derivatives was associated with a 2.0-fold increase in risk (Table 3). However, relatively few subjects had used these drugs (only 13 cases and six controls and all took phenylbutazone), and the relative risk estimate was not statistically significant.

### DISCUSSION

To our knowledge, the present study is the largest case–control study of bladder cancer ever conducted in a single, geographically defined study population. Our study was specifically designed to examine the association between analgesic use and bladder cancer, and detailed data were collected on this exposure. Our data demonstrate that sustained use of analgesics is not related to an increased risk of bladder cancer, as it is for cancer of the renal pelvis (McCredie et al, 1982) and parenchyma (Gago-Dominguez et al, 1999). In fact, compared with non- or irregular users, regular analgesic users were at an overall decreased risk of bladder cancer (OR = 0.81, 95% CI 0.68–0.96). However, there were clear differences in both the direction and strength of the associations between different classes of analgesics and bladder cancer risk. Intake of phenacetin was positively related to bladder cancer risk in a dose-dependent manner while intake of its major metabolite in humans, acetaminophen, was unrelated to risk. Intake of all classes of NSAIDs, except pyrazolon derivatives, were negatively associated with bladder cancer risk, with suggestive evidence that the protective effect varies in strength by subcategories of formulation. Acetic acids seemed to exhibit the strongest protective effect whereas aspirin/other salicylic acids and oxicam showed the weakest protection.

It is unlikely that recall bias could explain our results on NSAID use and bladder cancer. We have indirect evidence to support the absence of such a bias. A parallel case–control study of renal cell carcinoma that we conducted used the same set of medication questions as the current study as well as the same team of interviewers. Unlike the present results of a protective effect for cancer in NSAID users, the former study demonstrated statistically significant increased cancer risk among all categories of analgesic users (Gago-Dominguez et al, 1999). More importantly, the renal cell carcinoma study included a drug validation component in which all self-reported use of prescription analgesics was validated against physician/clinic records. Rates of concordance were remarkably similar between case and control groups, indicating a general lack of recall bias among cancer patients.

Chronic use of analgesics was first linked to the development of malignant growth in the urothelium through a series of case reports which documented cancer of the renal pelvis occurring in heavy users of phenacetin (Hultengren et al, 1965; Angervall et al, 1969). Johansson and Wahlqvist (1977) reviewed a series of case reports which documented bladder cancer among individuals with a history of phenacetin abuse. These uncontrolled observations were later followed by a few case–control studies (Fokkens, 1979; McCredie et al, 1983; Piper et al, 1985; McCredie and Stewart, 1988). Although fairly strong associations between regular use and increased risk were found in these studies, a dose–response relationship was demonstrated only in the Australian study (McCredie et al, 1983). Experimental studies have found phenacetin to cause bladder cancer in rats (Johansson, 1981; Murai et al, 1993). The present study confirms that sustained use of phenacetin is a bladder cancer risk factor, conferring a 1.5-fold excess risk among regular users of this drug. Phenacetin has been absent from all drugs manufactured in the USA since 1987.

Acetaminophen has been examined as a separate risk factor for bladder cancer in both an Australian and a US study (Piper et al, 1985; McCredie and Stewart, 1988). The results suggested that heavy use of acetaminophen-containing analgesics does not increase bladder cancer risk. A more recent case–control study (Derby et al, 1996) found a small, non-significant increased risk of bladder cancer among subjects with heavy acetaminophen exposure (OR = 1.3). Flaks et al (1985) found that long-term oral administration of acetaminophen induces transitional cell papillomas of the bladder, suggesting that acetaminophen could be a weak bladder carcinogen in this species, but Hiraga et al (1985) found no such evidence in a different rat species. Our data suggest that regular use of acetaminophen is not associated with the development of bladder cancer in humans.

The potential chemopreventive effects of aspirin on chemically-induced bladder cancer has been studied in rodents; results are mixed. Rao et al (1996) and Cohen et al (1989) noted no difference in incidence of bladder carcinomas between rats fed carcinogen only and those fed aspirin (up to 800 ppm in Rao et al and 5000 ppm in Cohen et al), concurrently with the carcinogen. On the other hand, Murasaki et al (1984) reported a statistically significant reduction in bladder cancer incidence in rats fed aspirin (5000 ppm) concurrently with the carcinogen relative to rats fed the carcinogen only (ten of 27 rats in former group had cancer vs 18 of 21 in latter group). Klän et al (1993) also observed a significant reduction in bladder cancer incidence among rats fed aspirin (1000 ppm) and carcinogen compared to rats fed carcinogen only (1/29 vs 8/29). Thus, there is suggestive experimental evidence that high-dose aspirin can protect against bladder cancer development.

There also exists experimental evidence in support of other types of NSAIDs as chemopreventive agents of bladder carcinogenesis. Acetic acids (indomethacin, sulindac) at dosage levels of 7.5 ppm or higher have been shown to reduce the incidence of carcinogen-induced bladder cancer by 70% or more (Grubbs et al, 1993; Shibata et al, 1993; Rao et al, 1996). Similarly, propionic acids (ketoprofen) and oxicams (piroxicam) reduce cancer incidence by comparable amounts (Moon et al, 1993; Rao et al, 1996). Our study supports these experimental findings.

In the current study, intake of pyrazolon derivatives unlike the other NSAIDs was associated with a substantial, although statistically non-significant, twofold increased risk of bladder cancer. Importantly there is experimental support for this epidemiological observation as well. Phenylbutazone, a pyrazolon derivative, induces transitional cell papillomas of the bladder in rats (Kari et al, 1995).

The present study provides the first detailed set of epidemiological data strongly suggesting that NSAIDs are protective against bladder cancer development. The large sample size allowed us

© 2000 Cancer Research Campaign 

to examine risk by categories of formulation. Excluding the pyrazolon derivatives acetic acids were the most effective and salicylic acids (including aspirin) as least effective in reducing bladder cancer risk. The only prior human data on this issue were from a large prospective study which examined aspirin use and risk of fatal cancer, in which no association was observed with mortality from cancers of the urinary tract overall (Thun et al, 1993).

Epidemiological studies have revealed that NSAIDs are promising candidates for chemoprevention against cancer of the colon. Experimental data are supportive of this hypothesis and also suggest that NSAIDs might provide protection against cancers of the mammary gland, skin and liver as well as the urinary bladder (Moon et al, 1992; Kelloff et al, 1994; Giardiello et al, 1995; Denda et al, 1997). The preventive mechanisms remain to be elucidated in detail but have been postulated to involve their inhibition of cyclooxygenase (COX), thereby inhibiting production of prostaglandins which influence tumour growth through both by stimulating cell proliferation and by disturbing immunological surveillance (Marnett, 1992; Giardiello et al, 1995; Lupulescu, 1996). There are two COX isoenzymes in humans, COX-1 and COX-2, and limited evidence suggests that COX-2 is the pivotal COX enzyme involved in carcinogenesis. COX-2, in contrast to the constitutively expressed COX-1 which contributes to physiological functions in most tissues, is inducible and has proven involvement in inflammatory responses and cell proliferation (Herschman, 1994). It is known that COX-2 is highly expressed in colon, stomach, skin and mammary tumours (Kargman et al, 1995; Müller-Decker et al, 1995; DuBois et al, 1996; Liu et al, 1996; Ristimäki et al, 1997), in conjunction with increased levels of prostaglandins (Lupulescu, 1996).

There is experimental evidence implicating the involvement of COX-2 in bladder cancer carcinogenesis. Okajima et al (1998) reported statistically significant, dose-dependent reductions (30–65%) in tumour incidence in nitrosamine-treated rats when the animals were administered a selective COX-2 inhibitor concurrently with carcinogen treatment.

This is the first epidemiological study to demonstrate a possible chemopreventive effect of long-term NSAID intake on bladder cancer development. Our observation has obvious public health significance, given that bladder cancer is the 11th commonest cancer worldwide, and the sixth commonest cancer in the USA (Kosary et al, 1995; Yu et al, 1998). Earlier, we had reported that sustained use of NSAID might contribute to the development of renal cell carcinoma, and that such exposure might explain approximately 20% of the cases diagnosed in Los Angeles County (Gago-Dominguez et al, 1999). On the other hand, there is growing evidence that NSAID use protects against colorectal cancer, Alzheimer's disease and ischaemic heart disease (Steering Committee of the Physicians' Health Study Research Group, 1989; Giovannucci et al, 1995; McGeer et al, 1996). This study suggests that NSAID use also may protect against another relatively common cancer in the USA as well as worldwide. If proven to be true, it will represent an important new dimension to the risk–benefit equation of NSAID use. One needs to bear in mind that while the incidence of renal cell carcinoma in US non-Hispanic Whites is about 6/100 000 (Yu et al, 1999), the comparable figures for bladder cancer, colorectal cancer, Alzheimer's disease and fatal ischaemic heart disease are 20, 44, 95 and 204 per 100 000, respectively (Kokmen et al, 1993; Liu et al, 1996; National Center for Health Statistics, 1996).

### ACKNOWLEDGEMENTS

We thank Ms Susan Roberts and Ms Kazuko Arakawa of the University of Southern California for their assistance in data collection and management. This study was supported by grants P01 CA17054, and R35 CA53890, and ROI CA65726 from the United States National Cancer Institute, and grant P30 E507048 from the United States National Institute of Environmental Health Sciences.

### APPENDIX

### Brand-name analgesics (over-the counter and prescription) categorized by formulation

*Aspirin only*: Momentum, regular aspirin, extra strength aspirin, buffered aspirin, arthritis strength Bufferin, regular Anacin, arthritis pain formula Anacin, Cama, 4-way cold tablet, Midol, Alka-seltzer, aspirin with codeine, Percodan, Equagesic, Talwin compound.
*Aspirin + Phenacetin*: APC, ASA compound, Empirin compound, Empirin with codeine, APC with codeine, Darvon compound, Fiorinal, Norgesic or Norgesic Forte, Phenaphen, Buff-a-comp, Synalgos.
*Aspirin + Acetaminophen*: extra strength Excedrin, Vanquish, Goody's headache powders.
*Aspirin + Other NSAID + Acetaminophen*: Excedrin.
*Aspirin + Other NSAID*: Stanback, BC powder or tablets.
*Acetaminophen only*: regular strength Tylenol, extra strength Tylenol, Cotylenol, generic acetaminophen, Anacin-3 regular strength or aspirin-free Anacin, Anacin-3 maximum strength or aspirin-free Anacin, Excedrin PM or aspirin-free Excedrin, regular strength Datril, extra strength Datril, Comtrex, Coricidin, Dristan or advanced formula Dristan, Nyquil, Robitussin night relief, Sine-aid, Sinutab, Triamicin, Bromoseltzer, Pamprin, Tempra, Tylenol with codeine, Tylox, Darvocet, Percocet-5, Midrin, Valadol, Percogesic.
*Acetaminophen + Phenacetin*: Repan.
*Acetaminophen + Other NSAID*: Bancaps, Arthralgen.
*Phenacetin only*: Wigraine.
*Phenacetin + Other NSAID*: Medache.
*Other NSAID only*: other salicylic acids: Doan's pill; propionic acids: Advil, Nuprin, Motrin, Anaprox, Naprosyn, Nalfon; acetic acids: Clinoril, Indocin; pyrazolon derivatives: Phenylbutazone; Oxicam: Feldene.
*Narcotic analgesics*: Darvon, Talwin or Talwin 50.

### REFERENCES

Angervall L, Bengtsson U, Zetterlund CG and Zsigmond M (1969) Renal pelvic carcinoma in a Swedish district with abuse of a phenacetin-containing drug. *Br J Urol* **41:** 401–405

Bernstein L and Ross RK (1991) *Cancer in Los Angeles County. A Portrait of Incidence and Mortality 1972–1987*. University of Southern California: Los Angeles, CA

Boring CC, Squires TS, Tong T and Montgomery S (1994) Cancer statistics, 1994. *CA Cancer J Clin* **44**: 7–26

Breslow NE and Day NE (1980) *Statistical Methods in Cancer Research, Vol. I: The Analysis of Case–Control Studies*. IARC Scientific Publication 32. IARC: Lyon, France

 *© 2000 Cancer Research Campaign*

Cohen SM, Hasegawa R, Sakata T and Johansson SL (1989) Effect of aspirin on urinary bladder carcinogenesis initiated with N-[4-(5-nitro-2-furyl)-2-thiazolyl] formamide in rats. *Cancer Res* **49**: 372–377
Denda A, Endoh T, Kitayama W, Tang Q, Noguchi O, Kobayashi Y, Akai H, Okajima E, Tsujiuchi T, Tsutsumi M, Nakae D and Konishi Y (1997) Inhibition by piroxicam of oxidative DNA damage, liver cirrhosis and development of enzyme-altered nodules caused by a choline-deficient, L-amino acid-defined diet in rats. *Carcinogenesis* **18**: 1921–1930
Derby LE and Jick H (1996) Acetaminophen and renal and bladder cancer. *Epidemiology* **7**: 358–362
DuBois RN, Radhika A, Reddy BS and Entingh AJ (1996) Increased cyclooxygenase-2 levels in carcinogen-induced rat colonic tumors. *Gastroenterology* **110**: 1259–1262
Flaks B, Flaks A and Shaw AP (1985) Induction by paracetamol of bladder and liver tumours in the rat. Effects on hepatocyte fine structure. *Acta Pathol Microbiol Immunol Scand [A]* **93**: 367–377
Fokkens W (1979) Phenacetin abuse related to bladder cancer. *Environ Res* **20**: 192–198
Gago-Dominguez M, Yuan J-M, Castelao JE, Ross RK and Yu MC (1999) Regular use of analgesics is a risk factor for renal cell carcinoma. *Br J Cancer* **81**: 542–548
Giardiello FM, Offerhaus GJ and DuBois RN (1995) The role of nonsteroidal anti-inflammatory drugs in colorectal cancer prevention. *Eur J Cancer* **31A**: 1071–1076
Giovannucci E, Egan KM, Hunter DJ, Stampfer MJ, Colditz GA, Willett WC and Speizer FE (1995) Aspirin and the risk of colorectal cancer in women. *N Engl J Med* **333**: 609–614
Grubbs CJ, Juliana MM, Eto I, Casebolt T, Whitaker LM, Canfield GJ, Manczak M, Steele VE and Kelloff GJ (1993) Chemoprevention by indomethacin of N-butyl-N-(4-hydroxybutyl)-nitrosamine-induced urinary bladder tumors. *Anticancer Res* **13**: 33–36
Herschman HR (1994) Regulation of prostaglandin synthase-1 and prostaglandin synthase-2. *Cancer Metastasis Rev* **13**: 241–256
Hiraga K and Fujii T (1985) Carcinogenicity testing of acetaminophen in F344 rats. *Jpn J Cancer Res* **76**: 79–85
Hultengren N, Lagergren C and Ljungqvist A (1965) Carcinoma of the renal pelvis in renal papillary necrosis. *Acta Chir Scand* **130**: 314–320
Johansson S and Wahlqvist L (1977) Tumours of urinary bladder and ureter associated with abuse of phenacetin-containing analgesics. *Acta Pathol Microbiol Scand [A]* **85**: 768–774
Johansson SL (1981) Carcinogenicity of analgesics: long-term treatment of Sprague-Dawley rats with phenacetin, phenazone, caffeine and paracetamol (acetaminophen). *Int J Cancer* **27**: 521–529
Kargman SL, O'Neill GP, Vickers PJ, Evans JF, Mancini JA and Jothy S (1995) Expression of prostaglandin G/H synthase-1 and -2 protein in human colon cancer. *Cancer Res* **55**: 2556–2559
Kari F, Bucher J, Haseman J, Eustis S and Huff J (1995) Long-term exposure to the anti-inflammatory agent phenylbutazone induces kidney tumors in rats and liver tumors in mice. *Jpn J Cancer Res* **86**: 252–263
Kelloff GJ, Boone CW, Crowell JA, Steele VE, Lubet R and Sigman CC (1994) Chemopreventive drug development: perspectives and progress. *Cancer Epidemiol Biomarkers Prev* **3**: 85–98
Klän R, Knispel HH and Meier T (1993) Acetylsalicylic acid inhibition of N-butyl-(4-hydroxybutyl) nitrosamine-induced bladder carcinogenesis in rats. *J Cancer Res Clin Oncol* **119**: 482–485
Kokmen E, Beard CM, O'Brien PC, Offord KP and Kurland LT (1993) Is the incidence of dementing illness changing? A 25-year time trend study in Rochester, Minnesota (1960–1984). *Neurology* **43**: 1887–1892
Kosary CL, Ries LAG, Miller BA, Hankey BF, Harras A and Edwards BK (eds) (1995) *SEER Cancer Statistics Review, 1973–1992: Tables and Graphs, National Cancer Institute*. NIH Pub. No. 96-2789, Bethesda, MD
Kune GA, Kune S and Watson LF (1988) Colorectal cancer risk, chronic illnesses, operations, and medications: case control results from the Melbourne Colorectal Cancer Study. *Cancer Res* **48**: 4399–4404
Liu L, Deapen D, Berstein L and Ross R (1998) *Cancer in Los Angeles County: Incidence and Mortality by Race/Ethnicity 1988–1995*. Los Angeles County Cancer Surveillance Program, University of Southern California
Liu XH and Rose DP (1996) Differential expression and regulation of cyclooxygenase-1 and -2 in two human breast cancer cell lines. *Cancer Res* **56**: 5125–5127
Lupulescu A (1996) Prostaglandins, their inhibitors and cancer. *Prostaglandins Leukot Essent Fatty Acids* **54**: 83–94
McCredie M and Stewart JH (1988) Does paracetamol cause urothelial cancer or renal papillary necrosis? *Nephron* **49**: 296–300
McCredie M, Ford JM, Taylor JS and Stewart JH (1982) Analgesics and cancer of the renal pelvis in New South Wales. *Cancer* **49**: 2617–2625
McCredie M Stewart JH, Ford JM and MacLennan RA (1983) Phenacetin-containing analgesics and cancer of the bladder or renal pelvis in women. *Br J Urol* **55**: 220–224
McGeer PL, Schulzer M and McGeer EG (1996) Arthritis and anti-inflammatory agents as possible protective factors for Alzheimer's disease: a review of 17 epidemiologic studies. *Neurology* **47**: 425–432
Marnett LJ (1992) Aspirin and the potential role of prostaglandins in colon cancer. *Cancer Res* **52**: 5575–5589
Moon RC, Detrisac CJ, Thomas CF and Kelloff GJ (1992) Chemoprevention of experimental bladder cancer. *J Cell Biochem Suppl* **161**: 134–138
Moon RC, Kelloff GJ, Detrisac CJ, Steele VE, Thomas CF and Sigman CC (1993) Chemoprevention of OH-BBN-induced bladder cancer in mice by piroxicam. *Carcinogenesis* **14**: 1487–1489
Müller-Decker K, Scholz K, Marks F and Fürstenberger G (1995) Differential expression of prostaglandin H synthase isozymes during multistage carcinogenesis in mouse epidermis. *Mol Carcinog* **12**: 31–41
Murai T, Mori S, Machino S, Hosono M, Takeuchi Y, Ohara T, Makino S, Takeda R, Hayashi Y, Iwata H, et al (1993) Induction of renal pelvic carcinoma by phenacetin in hydronephrosis-bearing rats of the SD/cShi strain. *Cancer Res* **53**: 4218–4223
Murasaki G, Zenser TV, Davis BB and Cohen SM (1984) Inhibition by aspirin of N-[4-(5-nitro-2-furyl)-2-thiazolyl] formamide-induced bladder carcinogenesis and enhancement of forestomach carcinogenesis. *Carcinogenesis* **5**: 53–55
National Center for Health Statistics (1996) *Vital Statistics of the United States, 1992: Vol. 2, Mortality, Part A* United States Department of Health and Human Services Publication PHS 96–1101. US Dept of Health and Human Services, Public Health Service, CDC: Hyattsville MD
Okajima E, Denda A, Ozono S, Takahama M, Akai H, Sasaki Y, Kitayama W, Wakabayashi K and Konishi Y (1998) Chemopreventive effects of nimesulide, a selective cyclooxygenase-2 inhibitor, on the development of rat urinary bladder carcinomas initiated by *N*-butyl-*N*-(4-hydroxybutyl) nitrosamine. *Cancer Res* **58**: 3028–3031
O'Neill GP, Mancini JA, Kargman S, Yergey J, Kwan MY, Falgueyret JP, Abramovitz M, Kennedy BP, Ouellet M, Cromlish W, et al (1994) Overexpression of human prostaglandin G/H synthase-1 and -2 by recombinant vaccinia virus: inhibition by nonsteroidal anti-inflammatory drugs and biosynthesis of 15-hydroxyeicosatetraenoic acid. *Mol Pharmacol* **45**: 245–254
Piper JM, Tonascia J and Matanoski GM (1985) Heavy phenacetin use and bladder cancer in women aged 20 to 49 years. *N Engl J Med* **313**: 292–295
Rao KV, Detrisac CJ, Steele VE, Hawk ET, Kelloff GJ and McCormick DL (1996) Differential activity of aspirin, ketoprofen and sulindac as cancer chemopreventive agents in the mouse urinary bladder. *Carcinogenesis* **17**: 1435–1438
Ristimäki A, Honkanen N, Jänkälä H, Sipponen P and Härkönen M (1997) Expression of cyclooxygenase-2 in human gastric carcinoma. *Cancer Res* **57**: 1276–1280
Shibata MA, Hasegawa R, Shirai T, Takesada Y and Fukushima S (1993) Chemoprevention by indomethacin of tumor promotion in a rat urinary bladder carcinogenesis model. *Int J Cancer* **55**: 1011–1017
Steering Committee of the Physicians' Health Study Research Group (1989) Final report on the aspirin component of the ongoing Physicians' Health Study. *N Engl J Med* **321**: 129–135
Thun MJ, Namboodiri MM, Calle EE, Flanders WD and Heath CW Jr (1993) Aspirin use and risk of fatal cancer. *Cancer Res* **53**: 1322–1327
Yu MC and Ross RK (1998) Epidemiology of bladder cancer. *Carcinoma of the Bladder*. *Innovations in Management*, Petrovich Z, Baert L and Brady LW (eds), pp. 1–13, Springer Verlag: Berlin, Heidelberg
Yu MC, Yuan JM and Ross RK (1999) Epidemiology of renal cell carcinoma. In: *Carcinoma of the Kidney, Testis and Rare Urologic Malignancies: Innovations in Management*, Petrovich Z, Baert L and Brady LW (eds), pp. 3–13. Springer-Verlag: Berlin, Heidelberg

© 2000 Cancer Research Campaign 
